# Novel Coronavirus disease (COVID-19) in newborns and infants: what we know so far

**DOI:** 10.1186/s13052-020-0820-x

**Published:** 2020-04-29

**Authors:** Domenico Umberto De Rose, Fiammetta Piersigilli, Maria Paola Ronchetti, Alessandra Santisi, Iliana Bersani, Andrea Dotta, Olivier Danhaive, Cinzia Auriti, The Study Group of Neonatal Infectious Diseases of The Italian Society of Neonatology (SIN)

**Affiliations:** 1 Neonatal Intensive Care Unit, Department of Neonatology, Bambino Gesù Children’s Hospital, IRCCS, Piazza S. Onofrio 4, 00165 Rome, Italy; 2 Division of Neonatology, St-Luc University Hospital, Catholic University of Louvain, 1200 Brussels, Belgium

**Keywords:** Newborns, COVID-19, Infants, SARS-CoV-2, Coronavirus

## Abstract

Recently, an outbreak of viral pneumonitis in Wuhan, Hubei, China successively spread as a global pandemia, led to the identification of a novel betacoronavirus species, the 2019 novel coronavirus, successively designated 2019-nCoV then SARS-CoV-2). The SARS-CoV-2 causes a clinical syndrome designated coronavirus disease 2019 (COVID19) with a spectrum of manifestations ranging from mild upper respiratory tract infection to severe pneumonitis, acute respiratory distress syndrome (ARDS) and death. Few cases have been observed in children and adolescents who seem to have a more favorable clinical course than other age groups, and even fewer in newborn babies. This review provides an overview of the knowledge on SARS-CoV-2 epidemiology, transmission, the associated clinical presentation and outcomes in newborns and infants up to 6 months of life.

## Introduction

Six coronavirus species were known so far to cause human disease, four of which (229E, OC43, NL63, and HKU1) typically cause cold symptoms in immunocompetent subjects [1]; the remaining two (Severe acute respiratory syndrome coronavirus – SARS-CoV – and Middle East respiratory syndrome coronavirus – MERS-CoV –) are zoonotic in origin and have been identified as causal agents of severe respiratory disease outbreaks from cross-species infections [2]. In December 2019, a cluster of patients with severe pneumonia of unknown etiology, epidemiologically linked to a seafood wholesale market in Wuhan (Hubei Province, China) led to the discovery of a novel, previously unknown coronavirus, initially designated 2019-nCoV: this agent represents the seventh member of the *coronaviridae* family that has the potential to infect humans [3]. Since its emergence in Wuhan, despite strong restrictions to limit domestic people circulation, the 2019-nCoV infection has rapidly spread across China and all over the world. This highly contagious and deadly virus was subsequently named *Severe Acute Respiratory Syndrome Coronavirus 2* (SARS-CoV-2) [4] and the related disease “Coronavirus Disease 2019” (COVID-19) [5]. On March 11th 2020, COVID-19 was pronounced as pandemic by World Health Organization (WHO) [6]. As of April 7, 2020, the number of confirmed cases was 1.250.000 worldwide (more than 180 countries involved), with 69.210 deaths resulting in a crude fatality rate of 5.5% at time of writing [7].

All age groups are susceptible, but people with comorbidities or the elderly are more likely to develop a severe form of the disease. Although children seem to have less severe clinical symptoms when infected [8], the potential harm of this novel disease remains largely unknown in neonates, especially in preterm infants. In the largest pediatric population-based study to date with 2143 cases, over 90% ranged from asymptomatic to moderate. However, the proportion of severe and critical cases was 10.6% under 1 year of age, compared to 7.3, 4.2, 4.1 and 3.0% among the 1–5, 6–10, 11–15 and > 15 year subsets, suggesting that infants may be at higher risk of severe respiratory failure than initially thought [9]. This review provides an overview of the current knowledge of this new pandemic viral infection in this fragile population.

## Methods

The literature search for this article was primarily Internet based, using PubMed and Google. A list of search terms and phrases was compiled to focus on the general topics of ‘neonates’ or ‘newborns’ and ‘COVID-19’ or ‘2019-nCoV’ or ‘SARS-CoV-2’, without imposing restrictions on date or year, locations, study design, study aim, or inclusion/exclusion criteria. We reviewed all the articles published till April 07th, 2020. Studies not written in English or Italian were withdrawn.

### SARS, coronaviruses and children

SARS-CoV-2 is a positive-sense, single-stranded RNA virus, belonging to the genus betacoronavirus, with a 88–96% sequence identity to three bat-derived SARS-like coronaviruses (bat-SL-CoVZC45, bat-SL-CoVZXC21, RaTG13) and to a coronavirus strains isolated in pangolins -a scaly, ant-eating mammal highly trafficked for its presumed medicinal virtues and clandestinely sold in live animal markets such as Wuhan- which possesses the same six key residues of the angiotensin converting enzyme 2 (ACE2) receptor binding domain as SARS-CoV-2 (see below). Neither bat- nor pangolin-coronaviruses sampled thus far have the polybasic sites characterizing SARS-CoV-2, and none have enough similarity to qualify as its direct progenitor, but the diversity of coronaviruses in animal species is largely undersampled and scarsely explored [10]). Thus, SARS-CoV-2 might have acquired its genomic features by evolutionary mutation in an animal reservoir and passed to human from droppings, contaminated food material or other in the market or surrounding regions of Wuhan. Alternatively, a more distant progenitor of SARS-CoV-2 might have passed to human and gradually acquired its pathogenic features through adaptation during successive, undetected human-to-human transmissions.

Severe acute respiratory syndromes associated with coronaviruses seem to have a children-sparing pattern, as suggested by the available literature on SARS-CoV (2003) and MERS-CoV (2012) epidemics [11–13]. Stockman et al. reported that children and adolescents are susceptible to SARS-associated coronavirus infection, although the clinical course and outcome are more favourable in children younger than 12 years of age compared with adolescents and adults: in fact no deaths were reported among children with SARS [11, 12]. Bartenfeld et al. reported that the only two fatal paediatric MERS-CoV cases had severe comorbidities (infantile nephrotic syndrome and cystic fibrosis) [13].

SARS- CoV-2 has a stronger transmission capacity than the former two, but the child-sparing trend seems to be similar, based on the data analysis regarding the early transmission dynamics of SARS-CoV-2 (December 2019–January 2020) on the first 425 confirmed cases in Wuhan: there were no severe cases in children below 15 years of age [14]. In the largest COVID-19 case series to date in mainland China (72,314 cases, updated through February 11, 2020), 416 cases (1%) had less than 10 years, but the incidence in neonates was not reported: however, no deaths occurred in this age group, while the overall case-fatality rate (CFR) was 2.3% [15].

The reason why children are less susceptible to COVID19 than adults remains unclear [16]. Children are not generally protected from viral infections, which instead are very frequent from infancy onwards. Otto et al. showed that children who had received combined diphtheria, pertussis, tetanus, HiB and poliomyelitis vaccination within the third month of life had significantly less symptomatic infections than those with delayed or partial immunizations, maybe because of an vaccination-associated unspecific enhancement of immunological activity (e.g. interleukin 2 mediated) or other factors still unknown [17]. A thorough understanding of the virus-host innate interactions during common respiratory infections of childhood (coronaviruses, rhinoviruses,, pneumoviruses (respiratory syncytial virus, metapneumovirus) and ortho/paramyxoviruses (influenza, parainfluenza), all characterized by an RNA genome, will be pivotal to understand these findings [18].

Another hypothesis involves the angiotensin-converting enzyme 2 (ACE2), a membrane-bound aminopeptidase highly expressed in lung alveolar epithelial cells and enterocytes of the small intestine, that plays a vital role in the cardiovascular and immune systems. ACE2 represents a major gateway to the SARS-CoV-2 infection, although the specific mechanisms are still uncertain. As with other coronaviruses, the N-terminal portion (S1, receptor binding domain) of the viral spike (S) glycoprotein binds with high-affinity to receptor(s) on the surface of susceptible cells. Although ACE2 has been identified as a viral receptor, there might be other receptors or co-receptors for this virus yet to be discovered [19]. It is possible that ACE2 tissue distribution differs between adults and children and the maturity and function (e.g., *binding ability*) of ACE2 in children may be lower than that in adults [9].

Moreover, children’s immune system is still developing: they are probably not yet able to start a cytokine storm similar to the adult one [20].

Furthermore, we cannot exclude that paediatric SARS-CoV-2 infections are often unrecognized or underestimated, as they may remain asymptomatic or manifest with mild, nonspecific symptoms such as hypo reactivity, headache, cough, nasal congestion, runny nose, and expectoration. Most children have only moderate to low-grade fever, or even none. Smaller infants can present primarily with gastrointestinal symptoms such as diarrhoea, abdominal distension and food aversion [21].

A subset of children presents with a mild respiratory failure, with hypoxia and increased respiratory rate unresponsive to standard oxygen therapy and requiring high-flow nasal cannulae or mask. Based on reported cases, most of these children belong to family clusters, and mostly of them have a benign course, usually recovering 1–2 weeks after disease onset. Newborns, in particular if preterm, need a more close and cautious observation, because they are more likely to present with insidious and non-specific symptoms as lethargy and even dehydration [3, 8, 9].

### Diagnosis

Human-to-human SARS-CoV-2 transmission has been demonstrated from subjects with or without clinical symptoms [22]. Transmission mainly occurs via droplets [14] but can also be through skin contact [23], faecal-oral transmission [24], and ocular surface contact [25]. The virus can be detected by Real Time Polymerase Chain Reaction (RT-PCR) in bronchoalveolar-lavage fluid [3], sputum [26], saliva [27] and, in particular, in nasopharyngeal swabs which are the gold standard for diagnosis [27]. The incubation period of COVID ranges from 2 to 14 days, though most cases declare between 3 and 7 days [8].

On laboratory examinations, leukopenia and lymphocytopenia are typical, while C-reactive protein and procalcitonin are usually within normal values. Other findings may include mild thrombocytopenia, and increased creatine kinase, alkaline phosphatase, alanine aminotransferase, aspartate aminotransferase, and lactate dehydrogenase [28].

Pulmonary lesions are shown more clearly by chest CT scan than X-ray examination; common findings include ground-glass opacity, multiple bilateral lobular and segmental consolidations, in particular in the peripheral lung [20].

Testing all admitted infants with respiratory symptoms for SARS-CoV-2 could represent a wrongful use of resources, since neonatal respiratory failure can result from a wide range of causes [29].

Infants should be suspected of SARS-CoV-2 if they are [28]:

- related with a cluster outbreak or exposed to relatives infected with SARS-CoV-2 (including family members, medical staff and visitors);
- delivered by suspected or confirmed SARS-CoV-2 infected mothers between 14 days before delivery and 28 days after delivery;
- showing lymphocytopenia or typical chest imaging findings.

### Maternal-foetal transmission and breastfeeding

Whether transmission can occur through mother–infant vertically or breast milk has not been clearly established yet. In a recent research letter, Dong et al. [30] speculate the possibility of the maternal fetal transmission of the virus. In a term infant, from mother with SARS-CoV-2 pneumonia from the 34th week of pregnancy, they found virus-specific IgG and IgM, high levels of Interleukin 6 and 10, negative nasopharyngeal swab and no symptoms, since 2 h from the delivery to the following 3 weeks. The authors conclude that despite uncertainty, so high IgM levels at 2 h of life would suggest an intrauterine infection. However, as Kimberlin and Stagno [31] commented, maternal-fetal transmission of the virus is possible, as its nucleic acid has been found in blood samples, but the only finding of specific IgM in the newborn is not enough and the probability of a false positivity is high.

There is currently no evidence showing vertical transmission and intrauterine SARS-CoV-2 infection in foetuses of women developing COVID-19 pneumonia in late pregnancy**.** Table 1 summarizes clinical characteristics of the cases described in literature to date [33–38], although data are available only on a small sample size. We included also an unpublished case of a preterm infant born to a mother with COVID-19, in Bruxelles, Belgium.

**Table 1:** Clinical features of neonates born to mothers with perinatal confirmed SARS-CoV-2 infection

	All	Dong et al. [30]	Chen et al. [32]	Li et al. [33]	Wang et al. [34]	Zhu et al. [35]	Chen et al. [36]	Liu et al. [37]	Zeng et al. [38]	Piersigilli et al. (unpublished)
Pregnant women	69	1	9	1	1	9 (1 was a mother of twins)	4	10	33	1
Caesarean section	53/69 (76.8%)	1	9/9	1	1	7/9	3/4	10/10	26/33	1
Positive maternal test (SARS-CoV-2 quantitative RT-PCR on samples from the respiratory tract)	68/69 (98.6%)	1	9/9	1	1	8/9 *	4/4	10/10	33/33	1
Median maternal age, years (range)	30 (22–40)	29	28 (26–40)	30	28	30 (25–35)	29.5 (23–34)	30.5 (22–36)	NA	37
Median gestational age (GA) on admission, weeks (range)	36 (26–39)	38	37 (36–39)	35	30	34 (31–39)	37.5 (37–39)	35.5 (32–38)	NA	26
Median onset of maternal symptoms to delivery, days (range)	2.5 (0–26)	26	3 (1–7)	2	6	2.5 (0–6)	NA	NA	NA	2
Maternal fever on admission	37/69 (53.6%)	1	7/9	1	1	8/10	3/4	8/10	8/33	0
Newborns	70	1	9	1	1	10	4	10	33	1
Positive cord blood samples	0	0 **	0/6	0	0	NA	NA	NA	NA	NA
Positive neonatal throat swab samples	4/63 tested	0 **	0/6	0	0	0/9;1 not tested	0/3; 1 not tested	0/10	3/33	1
Positive breastmilk samples	0	0	0/6	0	0	0/9; 1 not tested	0/3; 1 not tested	0/10	NA	0
Low birthweight (< 2500 g)	14/70 (20%)	0	2/9	NA	1	7/10	0	NA	3/33	1
Premature delivery	24/70 (34.3%)	0	4/9	1	1	6/10	1	6/10	4/33	1
Intrauterine foetal distress	15/69 (22%) + 1 stillbirth	0	0	1	6/10	1/4	3/10 + 1 stillbirth	0	2/33	2/9
Premature rupture of membranes	6/70 (8.6%)	0	2/9	0	0	3/10	0	1/10	3/33	0

*NA* not available

*The local CDC registered the mother of a couple of twins as a confirmed SARS-CoV-2 because of viral interstitial pneumonia at chest CT scan although RT-PCR returned negative

**In the newborn reported by Dong et al.,they found virus-specific IgG and IgM, high levels of Interleukin 6 and 10, negative nasopharyngeal swab and no symptoms, since two hours from the delivery to the following three weeks

We cannot definitively state whether caesarean section could prevent transmission from a pregnant mother with COVID-19 pneumonia more than vaginal delivery, because data are lacking.

However, maternal hypoxemia and fever caused by severe infection can lead to fetal distress, premature delivery and other risks [39]. Therefore, a detailed morphology scan at 18–24 weeks of gestation is indicated for pregnant women with suspected, probable or confirmed COVID-19 infection [40].

On February 6th, 2020, a neonate born by emergency caesarean section to a woman with COVID-19 pneumonia tested positive for SARS-CoV-2 infection 36 h after birth [32]. His mother had developed fever and was suspected to have COVID-19 pneumonia based on typical chest CT scan findings upon admission. The neonatal throat swab was obtained approximately 30 h after birth, thus providing no direct evidence for intrauterine infection.

The Belgian preterm infant included in Table 1 resulted positive for SARS-CoV-2 infection as well, but the pharyngeal swab was performed only at 6 days of life, after the finding of a viral pneumonia in mother.

Very recently Zeng et al. [38] reported a series of 33 infants from mothers with COVID-19: three of whom were symptomatic (one preterm with a gestational age – GA – of 31 weeks), with a radiological picture of pneumonia. Nasopharyngeal and rectal swab test yielded positive for SARS-CoV-2 virus. These infants presented a clinical course worse than the other ones described to date. The outcome was however favorable, with the test negativity at 6th and 7th days after the onset of symptoms in two infants and in the third, respectively. The 31 weeks neonate developed coagulopathy, associated with Enterobacter sepsis, which resolved with the recovery of sepsis. None of the newborn babies died.

No cord blood, amniotic fluid or placenta tests were performed, so whether the COVID-19 infection was acquire by intrauterine transmission or postnatally cannot be determined in these cases. Nevertheless, these five cases call for special protection measures in order to prevent postnatal infections in neonates born to mothers with COVID-19 pneumonia.

The eventuality of the vertical transmission of SARS-CoV-2 through breast milk could not be confirmed. Only limited data on SARS-CoV-2 excretion in breast milk are available: Chen et al. reported that all breast milk samples from 9 mothers with COVID-19 pneumonia were negative [32]. This is the reason why most neonatal guidelines do not recommend against breastfeeding from mothers with COVID-19. Nevertheless, specific precautions have to be taken, such as wearing a mask during breastfeeding and observing meticulous hand hygiene.

### Clinical features in newborns and infants

Clinical features of infected newborns, especially preterm infants, might be non-specific and include acute respiratory distress syndrome, temperature instability, gastrointestinal and cardiovascular dysfunction. All infants with suspected COVID-19 should be isolated and monitored, whether symptomatic or not [41].

In the cohort described by Zhu et al. [35], nine of ten neonates born to mothers with confirmed COVID-19 presented with shortness of breath (*n* = 6), cyanosis (*n* = 3), vomiting and feeding intolerance (*n* = 2), fever (n = 2), increased heart rate (*n* = 1), moaning and rashes (n = 3). Two of these newborns had thrombocytopenia accompanied by abnormal liver function. One of them died (a male with a GA of 34 + 5 weeks and a birthweight of 2200 g) because of gastric bleeding and shock resulting in multiple organ failure and disseminated intravascular coagulation despite blood transfusions and supportive treatment. Another patient (a female with a GA of 34 + 6 weeks and a birthweight of 2300 g) presented with gastrointestinal haemorrhage and disseminated intravascular coagulation but responded to gamma globulin intravenous administration and survived. Notably, none of these neonates tested positive for SARS-CoV-2.

In the cohort reported by Chen et al. [36] three of the four infants tested negative for SARS-CoV-2 using a throat swab specimen and one couldn’t be tested because of parental refusal: two of the four infants were healthy. Two of the four infants had rashes after birth, however, the rash distribution and shape differed (respectively, some maculopapules scattered all over the body and small military red papules on the forehead). One infant had mild post-natal dyspnea diagnosed as transient tachypnea of the newborn (TTN); he required just nasal-Continuous Positive Airway Pressure (nCPAP) for 3 days.

In the cohort described by Zeng et al. [38], among three positive newborns, the preterm neonate (GA 31 weeks) required mechanical ventilation.

No symptomatic neonates born to mothers with confirmed COVID-19 were described by Chen [32], Li [33] and Wang [34].

Li et al. compared clinical characteristics, maternal and neonatal outcomes of pregnant women with or without COVID-19 pneumonia: COVID-19 infection was not found in the newborns and none developed severe neonatal complications [42].

Clinical features of infants up to 6 months of life with confirmed postnatal SARS-CoV-2 infection reported in literature until now [43, 44] were summarized in Table 2: all were hospitalized but none required intensive care. The infant described by Kam et al. was brought to hospital for clinical assessment and isolation considering his close contact with confirmed COVID-19 cases, but remained asymptomatic [44].

**Table 2:** Clinical features of infants with a confirmed COVID-19

Patient	1	2	3	4	5
Reported in:	Wei et al. [43]				Kam et al. [44]
Age	1 month 26 days	3 months	3 months 22 days	6 months	6 months
Sex	Female	Female	Female	Male	Male
Country	China	China	China	China	Singapore
Symptoms at onset	Runny nose; cough	Cough; sputum production	Fever	NA	None
Time between admission and diagnosis (days)	1	1	1	2	NA
Epidemiologic history: no. of family members infected	2	2	1	1	3
Linkage to Wuhan	Yes	Yes	NA	No	Yes
Mechanical ventilation	No	No	No	No	No
Severe complications	No	No	No	No	No

*NA not available

The first three newborns with COVID-19 (age: 17 days, 5 days, 30 days), reported in a short review by Lu et al. [45] were not included in this table because described in Chinese: they all presented with shortness of breath, milk intolerance and fever; their vital signs were stable and symptoms were mild.

Newspapers reported other cases of infants with COVID-19 in Northern Italy in March 2020 without need for intensive care; they were not included in the table as they were not yet reported in the medical literature.

### Infection control and treatment

When handling suspected or confirmed infected mothers, the delivery room or operating room should be specially prepared, preferably with negative pressure when available [46]; physicians should wear adequate personal protective equipment (PPE). If the newborn is asymptomatic after birth and the suspected mother’s test is negative for SARS-CoV-2, the newborn can room in with the mother; if the maternal nasal swab is positive for SARS-CoV-2 and maternal quarantine is required, strict droplet/contact precautions are required. Many guidelines recommend the newborn to be isolated in a designated COVID-19 neonatology unit until cleared, although some allow rooming-in in mother’s room with due infection control measures (such as 2-m distance from cot to bed and droplet/contact precautions while breastfeeding). A SARS-CoV-2 test should be performed after birth: all probable or laboratory-confirmed newborns with SARS-CoV-2 should be isolated or cohorted in a single room (if possible) for at least 14 days. The quarantine room should be equipped with an isolated air cycle system and, given SARS-CoV-2 high infectivity, negative pressure isolation rooms are recommended, albeit rarely available [41]. Standard and additional infection control measures should be implemented immediately [47], such as visitor restriction policies and protected pathways.

If a newborn develops COVID-19 manifestations during the isolation period or is highly suspected of SARS-CoV-2 infection from admission, the patient should be immediately directed to a Neonatal Intensive Care Unit (NICU) of a reference hospital or to a designated COVID-19 pediatric unit.

Triage procedures should be implemented, as it is important to reserve NICU beds for patients who are in life-threatening situations [29], and an indiscriminate NICU admission policy might lead to mistakes in epidemiological data and overestimation of the severity of the disease in this category.

In case of neonatal transport, the transport incubator and the ambulance should be extensively disinfected after the transfer; strict droplet/contact precautions and isolation should be applied throughout the transport.

General management requires a strict continuous monitoring (heart rate, respiratory rate, oxygen saturation, temperature, blood pressure, blood glucose, and gastrointestinal symptoms), blood examinations and a chest X-ray. If necessary respiratory support should be delivered via High-flow nasal cannula (HFNC), non-invasive ventilation or mechanical ventilation. Antibiotics should be prescribed only to patients with probable or confirmed bacterial infection; empirical use should be avoided. Surfactant replacement therapy, inhaled nitric oxide (iNO), and high-frequency oscillatory ventilation (HFOV) might be effective for newborns with severe acute respiratory distress syndrome but no evidence-based data exist. The question of antenatal corticosteroids for induction of foetal lung maturation in COVID-19 -positive or -suspected mothers with preterm labor or predicted preterm delivery is controversial, since there is strong evidence that their use prevents death and improve long-term outcomes across the range of prematurity [48]; conversely, corticosteroids have shown no proven benefit and/or potential harm in other similar viral infections such as SARS, MERS, RSV and influenza [49]. In the absence of uniform guidelines to date, the risk/benefit ratio should be carefully weighed on an individual basis and take informed parental choices into account. For all procedures that may produce aerosols on suspected or confirmed infants, airborne precautions should be taken. In critically ill neonates, continuous renal replacement (CRRT) or extracorporeal membrane oxygenation (ECMO) could become necessary.

There are no data on effective antiviral drugs (ie remdesivir or lopinavir/ritonavir) and anti-cytokine storm (ie tocilizumab) for children with COVID-19 to date.

Intravenous immunoglobulin can be used in severe cases when indicated (1 g/kg/day for 2 days, or 400 mg/kg/day for 5 days), but its efficacy needs further evaluation [8].

Newborns could be discharged after resolution of respiratory symptoms, lack of fever for at least 3–5 days, and after two nasal swabs negative for SARS-CoV-2 taken at least 48 h apart [47].

As maternal separation may cause anxiety and maternal depression, psychological comfort should be offered to the parents [40].

## Conclusions

To our best knowledge, this review is the largest to date describing clinical features of SARS-CoV-2 infection in neonates and infants up to 6 months of age. Although we still do not know how massively SARS-CoV-2 will spread around the world and affect the population worldwide, the initial data reported in children so far are reassuring. However, due to the limited number of cases and clinical evidence, pediatricians should continually update their knowledge and be aware of the risks in particular in the high-risk population of newborn and preterm infants.

## Data Availability

The literature search for this article was primarily Internet based, using PubMed and Google.

## Abbreviations

ACE2: Angiotensin converting enzyme 2
ARDS: Acute respiratory distress syndrome
CFR: Case-fatality rate
COVID19: Coronavirus disease 2019
CRRT: Continuous renal replacement
nCPAP: Nasal-Continuous Positive Airway Pressure
ECMO: Extracorporeal membrane oxygenation
GA: Gestational age
HFNC: High-flow nasal cannula
HFOV: High-frequency oscillatory ventilation
MERS-CoV: Middle East respiratory syndrome coronavirus
NICU: Neonatal Intensive Care Unit
iNO: Inhaled nitric oxide
PPE: Personal protective equipment
RSV: Respiratory Syncitial Virus
RT-PCR: Real Time Polymerase Chain Reaction
SARS-CoV: Severe acute respiratory syndrome coronavirus
TTN: Transient tachypnea of the newborn
WHO: World Health Organization
